# Editorial: Optimization of spine surgery outcomes in the pre-, peri-, and postoperative settings

**DOI:** 10.3389/fsurg.2023.1235095

**Published:** 2023-08-21

**Authors:** Jonathan J. Rasouli, Jeremy Steinberger

**Affiliations:** ^1^ Neurosurgery, Northwell Health, New York, NY, United States; ^2^ Department of Neurosurgery and Orthopedics, Icahn School of Medicine at Mount Sinai, New York, NY, United States; ^3^ Director, Minimally Invasive Spine Surgery, Department of Neurosurgery, Mount Sinai Health Systems, New York, NY, United States

**Keywords:** optimization, spine, outcomes, ambulation, physical therapy

**Editorial on the Research Topic** Optimization of spine surgery outcomes in the pre-, peri-, and postoperative settings

Patient optimization is one of the strongest predictive indicators to improve outcomes and mitigate complications after spinal surgery. While we have collectively known that optimizing a patient's cardiopulmonary and metabolic status are essential prior to any surgical procedure, it has been challenging to develop a set of clinical decision rules to help guide spine patients. Hence, the development of enhanced recovery after surgery (ERAS) protocols to standardize optimization across surgeons and healthcare systems (1).

The importance of pre-operative optimization was recently highlighted by Maitra et al. who demonstrated patients who medically optimized prior to spine surgery demonstrate improvements in satisfaction scores, decreased complications, and decreased length of stay (2). The results of this study were further supported with several reviews demonstrating reduction in the risk of surgical site infection with the implementation of pre-operative optimization protcols (3, 4). Patient-specific modifiable risk-factors that are potential areas for focused intervention include strict blood glucose control, weight loss, smoking cessation, optimization of bone quality in patients with osteoporosis, opioid weaning, and optimizing mental health disorders (5). Identifying and treating these pre-operative co-morbidities can directly improve outcomes after spinal surgery.

Interventions aimed at enhancing intra- and post-operative optimization are essential, as well. For example, the use of intravenous tranexamic acid to mitigate blood loss, patient positioning to reduce intra-orbital pressure while prone, the use of intraoperative cell salvage, and blood pressure control when operating on or near the spinal cord have been shown to improve outcomes after spinal surgery (6–8). While intra-operative optimization is generally heterogenous and can significantly vary among surgeons, post-operative protocols such as early ambulation, physical therapy, blood glucose monitoring, and reduced opioid use have been shown to clearly correlate with improved outcomes in patients undergoing lumbar fusion surgery (9). Of note, these protocols guiding pre-, intra-, and post-operative optimization are interconnected and focusing on all phases of the surgical process is critical to optimize outcomes.

Clinicians should make every effort to “optimize” our patients before, during, and after spinal surgery. While more prospective and larger population studies are required, there is a strong consensus that optimization is as important as patient selection, decision-making, and surgical technique in guiding post-operative outcomes (10).
